# A Novel Prehydrated Porcine-Derived Acellular Dermal Matrix: A Histological and Clinical Evaluation

**DOI:** 10.1155/2024/7322223

**Published:** 2024-06-27

**Authors:** Andreas van Orten, Werner Goetz, Hakan Bilhan

**Affiliations:** ^1^ Private Dental Practice Do24, Dortmunder Str. 24–28, 45731 Waltrop, Germany; ^2^ Policlinic of Orthodontics Centre for Dental Care Basic Science Research in Oral Biology Friedrich-Wilhelms University, Welschnonnenstr. 17, 53111 Bonn, Germany; ^3^ Department of Periodontology School for Health Sciences Witten/Herdecke University, Alfred-Herrhausen-Str. 45, 58448 Witten, Germany

## Abstract

It is well known that soft tissue quality and quantity around dental implants is of paramount importance for later peri-implant health. For this purpose, the clinical and histological outcomes of the peri-implant mucosa, following soft tissue augmentation for soft tissue improvement with a novel prehydrated porcine acellular dermal matrix graft (PPADMG) in conjunction with simultaneous implant placement, were evaluated in this case series. Twenty-two patients were included in the study. They underwent a late implant placement protocol combined with PPADMG for soft tissue augmentation. A punch biopsy was taken at the time of uncovery of the submerged healed implant after a mean of 157 days healing time. Supracrestal soft tissue height (STH) was measured at the time of implant placement and uncovery. All sites showed a clinical increase in STH. The histological structure of the biopsies resembled a similar structure as found in the healthy oral mucosa. No unexpected tissue reactions could be found. Within the limits of this clinical and histological study, it may be concluded that STH improvement with this novel porcine-derived acellular dermal matrix, in combination with simultaneous implant placement, is a viable option to create a peri-implant tissue thickness and stability.

## 1. Introduction

In addition to the application in dentistry [1–3], the use of acellular dermal matrix graft (ADMG) has been described in many different ways since the 1990s: treatment of burn injuries [4], reconstructive and aesthetic breast reconstruction [5], dural replacement [6], ophthalmic plastic and reconstructive surgery [7], abdominal hernia repair [8], orthopedic surgery [9, 10], and healing of the skin in general [11]. Not only the substitution of autologous tissues but also the combination of autologous grafts with dermal matrix grafts appears promising in reconstructive surgery in order to decrease surgical invasiveness and increase patients' quality of life [12]. The use of dermal matrices as a biocontainer for biofunctionalization [13] in combination with, e.g., enamel matrix derivates, recombinant bone morphogenetic proteins [14], or autologous blood derivates [15] is a current object of research.

A well-established indication for the use of ADMGs for many years has been the coverage of gingival recessions in teeth with a thin phenotype. This can be achieved using both tunneling techniques and various coronally advanced flap procedures. Compared to autologous connective tissue graft (CTG), ADMGs tend to have the potential disadvantage that, after a healing period of 6 months, no further gain in keratinized mucosa is expected, whereas such gains can be observed over longer periods with CTG. In terms of soft tissue thickening, volume stability, and percentage of root coverage, there is no significant difference between CTG and ADMG [3].

Processed collagen membranes (CM) represent an alternative to ADMGs. They can be used for the coverage of gingival recessions as well as for the modification of soft tissues at implant insertion sites [16]. Their use as biocontainers also appears to be sensible [14, 17]. The manufacturing processes and underlying technologies are diverse, as are their mechanical capabilities and properties [18].

Especially for the creation of a zone of keratinized mucosa around implants, selected collagen membranes appear to be a good alternative to autologous tissues [19] and open healing of certain collagen membranes seems to be possible with minimal complications [20].

Different manufacturing processes and formulations (e.g., dry or prehydrated) of ADMGs could make a difference in clinical performance. Currently, the data are not sufficient to identify a preference in all aspects. Both forms show clinical improvement in terms of soft tissue thickening around implants [21].

Although the insertion of dental implants is an established and well-studied therapy option for the replacement of teeth and the stabilization of dentures [22], many implant-related biological and technical complications have been reported over time [23, 24]. Soft tissue thickness has been identified as one of the key factors in preventing marginal bone loss after implant placement, among other factors such as compromised patient health, malpositioning of implants, peri-implantitis, mechanical overload, and systemic diseases [25, 26]. It could be shown that utilizing bone level fixtures in patients with thin soft tissue (≤2 mm) is associated with an increased probability of visibility of the implant, or its prosthetic parts, through the mucosa than in a comparable patient group with thicker soft tissue (>2 mm) [27, 28]. Furthermore, thin soft tissue around dental implants resulted in greater marginal bone loss over time and thus is a crucial factor for mucositis or peri-implantitis [25, 29]. A correlation between soft tissue height and soft tissue thickness was demonstrated, with thickness appearing to be greater than height [30, 31]. Compromised aesthetic situations may also occur without marginal bone loss (MBL) due to midbuccal soft tissue recessions with a higher likelihood in combination with thinner peri-implant tissue [32]. The ideal mucosa thickness has been a subject of debate. Nowadays, there is evidence that the threshold for a positive prediction of less marginal bone loss, after implant placement, is an STH of approximately 3 mm or more [16, 33]. A reasonably thicker mucosa does not seem to have any disadvantage for the development of the emergence profile [34]. Different materials to increase soft tissue volume have been described: autologous connective tissue grafts (CTG), acellular dermal matrix grafts (ADMG), and collagen membranes (CM) [35], with both CTG and ADMG seeming to be superior in terms of soft tissue volume gain [36]. In some recently published studies, no significant difference in PES (pink esthetic scores) [37], volume gain [38, 39], or keratinized mucosa width gain was demonstrated between CTG and ADMG [39], but the available data are still inconclusive, especially in terms of volume gain and gain of keratinized mucosa [40]. The presence of a less than 2 mm zone of keratinized peri-implant mucosa (KPIM), or its absence, is associated with a higher frequency of clinical signs of inflammation and MBL [41, 42]. The standard of care to establish a band of attached and keratinized peri-implant mucosa (KPIM) is the augmentation of a free gingival graft (FGG) in combination with an apically positioned flap (APF), but like before data are inconclusive: Augmentation with soft tissue substitutes of xenogeneic origin may result in no significant differences [26]. Some studies show that patients can benefit in terms of less painful treatment when soft tissue substitutes are used [38, 43, 44], while other studies do not confirm these results and no significant difference can be observed between the two groups [45, 46].

The purpose of this case series was to assess the capacity and the clinical feasibility of a novel PPADMG to increase the STH in combination with simultaneous implant placement to prevent consecutive peri-implant marginal bone loss (MBL), as well as implant and abutment transparency. To study matrix regeneration and membrane degradation, biopsies were studied histologically. In addition, immunohistochemistry was applied to analyze matrix turnover and vascularization using markers which must be turned out to be useful to evaluate the healing of biomaterials [47].

## 2. Materials and Methods

Twenty-two subjects with a mean age of 58,26 years (range 31–77 years), 11 male/11 female, with 27 implant sites (late implant placement protocol) were included in this case series. The patients met the following inclusion criteria: older than 18 years, no medical history that contraindicates the surgical procedure, and at least one implant site with a STH ≤ 2.5 mm and a KMW ≥ 5 mm before implantation and soft tissue augmentation. Patient recruitment has taken place between October 2019 and April 2022. They had no peri-implantitis and no stage I and grade A periodontitis. All patients were participating in regular recalls with semiannual clinical check-ups and oral hygiene instructions.

The exclusion criteria were systemic diseases that might impair bone metabolism, antiresorptive therapy (as bisphosphonates), pregnancy and nursing period, psychiatric conditions, and oncologically relevant diseases. Smokers and patients with diabetes mellitus were not excluded. Three patients were cigarette smokers with a daily consumption of between 10 and 15 cigarettes. Five patients were former cigarette smokers, and fourteen patients were nonsmokers.

**Figure 1:**
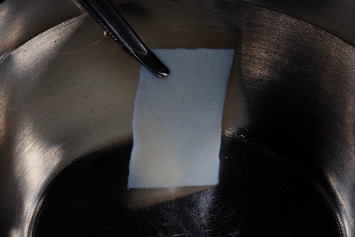
Native prehydrated porcine-derived acellular dermal matrix before implantation.

**Figure 2:**
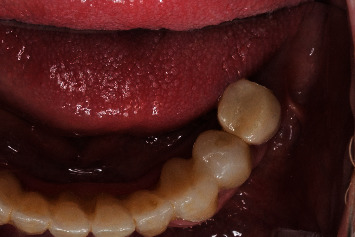
Regio #19-occlusal view.

**Figure 3:**
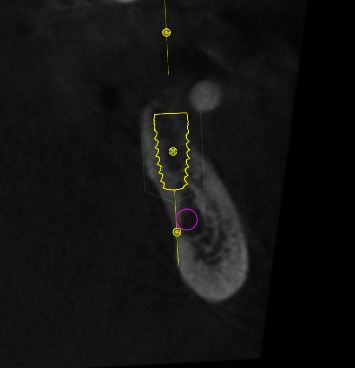
Regio #19-preoperative cross-sectional image from CBCT with visualization of the planned implant position and the inferior alveolar nerve.

**Figure 4:**
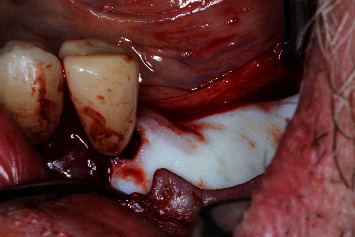
Regio #19-prehydrated porcine-derived acellular dermal matrix graft in situ to increase supracrestal tissue height and mucosa thickness.

**Figure 5:**
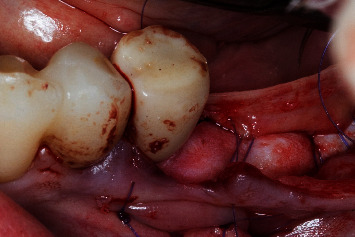
Regio #19-horizontal mattress suture to ensure immobility of the prehydrated porcine-derived acellular dermal matrix graft.

**Figure 6:**
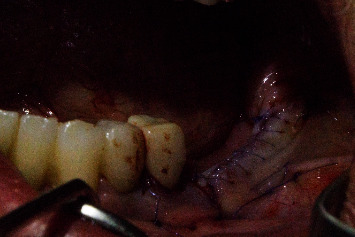
Regio #19-wound closure with complete coverage of the prehydrated porcine-derived acellular dermal matrix graft.

**Figure 7:**
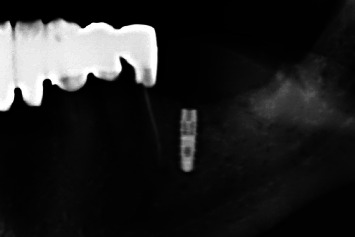
Regio #19-postoperative X-ray control (detail from panoramic tomogram).

Two hyperglycemic patients were enrolled in the study group (Pat. #11, male, 56 y, Hb1Ac = 6.5%; Pat. #15, male, 63 y, Hb1Ac = 7.5%). Each patient agreed to participate in the study, providing written informed consent. The scope of treatment followed the standard protocol of this practice. The Ethics Committee of the University of Bonn had approved the study protocol (ethical committee decision #222/05). All interventions and follow-up examinations were performed by the same practitioner with oral surgery experience of 20 years. Thirty minutes before the operation, oral antibiotic prophylaxis was administered (2 × 1 g tablet, amoxicillin, Aliud Pharma GmbH, Laichingen, Germany). Following infiltration anesthesia with Ultracain (UDS 1 : 200,000, Sanofi, Paris, France) at a dose of 1 ml per implant site, both vestibular and oral in the maxilla, and an additional block anesthesia of 1.8 ml in the mandible, a midcrestal incision was made using a 15C blade (Hu-Friedy, Chicago, IL, USA). Care was taken to ensure that the incision was made within the keratinized gingiva. In edentulous spaces, the incision was extended by one tooth width to enhance tissue mobilization, and in fully edentulous jaw sections, the incision was extended by 15 mm. Using a microelevator, a careful mobilization of the flap was performed, creating a mucoperiosteal flap with an extension of 5 mm vestibularly and orally, allowing for the preparation of the implant osteotomy without damaging the soft tissues. On the vestibular flap side, a periosteal release was performed, which, in the mandibular posterior region, was supplemented by the preparation of the lingual tissues, including the release of the superficial parts of the mylohyoid muscle. The STH was measured by means of NC12 periodontal probe (Colorvue PCVUNC12PT, Henry Schein, Melville, NY, USA). The initial 2 mm pilot implant drilling was performed using a surgical guide fabricated with the aid of CBCT scans (Orthophos 3D, Dentsply Sirona, York, PA, USA) and intraoral scans (Primescan, Dentsply Sirona, York, PA, USA) along with the software RealGUIDE 5.0 (3diemme, Cantù, Italy). Subsequent drilling was carried out using Densah Burs (Versah, Jackson, MI, USA), following the manufacturer's protocol for the respective implant. All implants were inserted at bone level and sealed with a cover screw. Prior to placement and according to the manufacturer's instructions, the PPADMG, which is delivered in an aqueous phosphate-buffered solution (NovoMatrix, LifeCell Corporation, Branchburg, USA) (Figure 1), was placed into a sterile basin and covered with room temperature sterile saline solution for a minimum of 2 minutes. Whenever guided bone regeneration (GBR) procedures were required, a collagen membrane (CM) (Mem-Lok RCM, BioHorizons, Birmingham, AL, USA) was used over the bone substitute materials (BSM). When autologous bone was used, the dermal matrix was placed directly on the autologous bone. It was prepared in one or two layers over the implants (Figures 2–5). Subsequently, the PPADMG was fastened to the genuine underlying bone, the autologous graft or the BSM in combination with the CM by one or more absorbable horizontal deep mattress sutures (6/0 Monofast, Medipac Manufacturing, Stavrochori-Kilkis, Greece) (Figure 3). Finally, the midcrestal incision was sutured with 6/0 Glycolon violet single interrupted or continuous interlocking sutures (Resorba Medical GmbH, Nurnberg, Germany) (Figure 6). Postoperative evaluation included radiographic assessment to verify the correct positioning of the implant (Figure 7). Postoperatively, the patient was provided with nonsteroidal anti-inflammatory drugs for analgesia (800 mg ibuprofen, Ibuflam, Zentiva, Pharma GmbH, Berlin, Germany, every 8 hours on demand) and antibiotics for infection control (1 g Amoxicillin, every 8 hours for 7 days). The postop regimen also included the patient's instruction to abstain from mechanical plaque control in the treated area for one week and use chlorhexidine (Chlorhexamed GlaxoSmithKline Consumer Healthcare GmbH and Co. KG, Munich, Germany) mouth rinse (0.2%) twice a day instead. The patients were provided with a mobile emergency contact number. However, no patient needed to use it due to unforeseen complications. The first postoperative check-up was performed after 24 hours.

**Figure 8:**
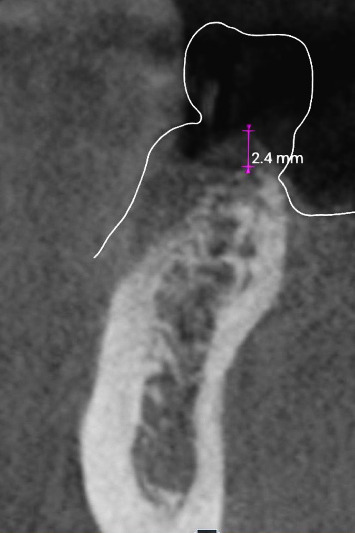
Regio #30-cross-sectional image from CBCT with visualization of the soft tissue height before implantation.

**Figure 9:**
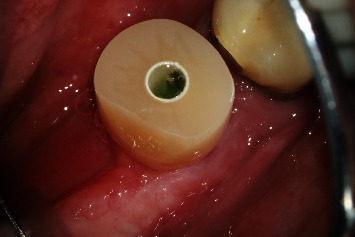
Regio #30 after placement of individual healing abutment.

**Figure 10:**
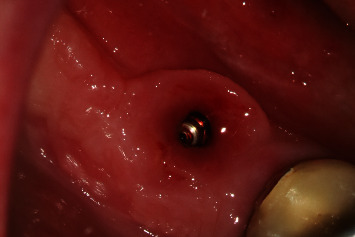
Regio #30 after removal of individual healing abutment.

**Figure 11:**
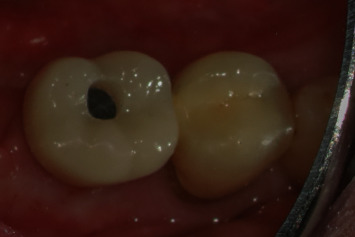
Regio #30 with screw-retained superstructure.

**Figure 12:**
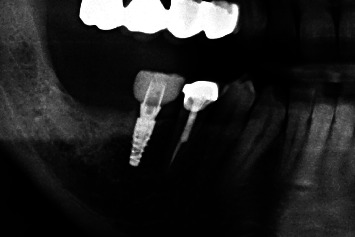
Regio #30-postoperative X-ray control (detail from panoramic tomogram).

The next follow-up appointments were scheduled for ten days and four weeks postoperatively. During these visits, the single interrupted sutures were removed after 10 days, and the horizontal mattress sutures were removed after 4 weeks. In some cases, the mattress sutures could not be removed because they tore at the knot during removal. A healing time of at least 8 weeks was intended to provide a solid implant site. After applying 1.8 ml of a local anesthetic (Ultracain DS forte, Sanofi, Paris, France), the implant site was prepared by means of the previously applied surgical stent in combination with a gingival punch (J5041.3303, Camlog GmbH, Wimsheim, Germany) to biopsy and gain access to the implant's cover screw at the same time. By analogy with the first surgery, the STH was measured again by means of a NC12 periodontal probe (Colorvue PCVUNC12PT, Henry Schein, Melville, NY, USA). Subsequently, the intended healing abutment was placed. After carefully removing the biopsy from the punch, it was stored in a 10% buffered formalin solution. A conventional impression was taken after 4 weeks, and the insertion of the final restoration took place after a further 4 weeks (Case 2, Figures 8–12).

### 2.1. Histological Analysis

Each biopsy sample was fixed by immersion in 4% buffered formaldehyde (Sörensen buffer) at room temperature (RT) for at least 1 d and subsequently decalcified for about 2 to 3 weeks in 4.1% disodium ethylenediaminetetraacetic acid (EDTA) solution, which was changed every 24 h, due to probable remnants of bone substitutes in the subepithelial layers. After hydration, tissues were dehydrated in an ascending series of ethanol and embedded in paraffin. Serial sections of 2-3 *μ*m were cut and representative slides were stained with hematoxylin-eosin (HE), Masson–Goldner, and PAS (periodic acid Schiff).

### 2.2. Immunohistochemistry

Representative slides from the median parts of the sample series were deparaffinized, rehydrated, and rinsed for 10 min in Tris-buffered saline (TBS). Endogenous peroxidase was blocked in a methanol/H_2_O_2_ (Merck, Darmstadt, Germany) solution for 45 min in the dark. Sections were pretreated with PBS containing 1% bovine serum albumin (BSA) for 20 min at RT, digested with 0.4% pepsin for 10 min at 37°C, and afterwards incubated with the primary antibodies in a humid chamber. The following markers were investigated: for extracellular matrix collagen type I (COL1) and osteopontin (OP) and for vessels von Willebrand factor (vWF). Antibody details and incubation protocols are listed in Table 1.

Detection of antibody binding was performed with EnVision® anti-rabbit HRP-conjugated secondary antibodies (Dako, Glostrup, Denmark), diluted 1 : 50, and incubated for 30 min at RT. Peroxidase activity was visualized using diaminobenzidine (DAB) yielding a brown staining product and slides were counterstained with Mayer's hematoxylin. Specificity controls (not shown) were run by (i) omitting primary antibodies and applying TBS or normal horse serum instead and (ii) omitting primary antibodies or bridge and secondary antibodies, respectively.

## 3. Results

### 3.1. Patient Demographics and Clinical Data

The study included 27 implant sites in 22 patients with a mean age of 58.26 years (range 31–77 years), including 11 males and 11 females. Table 1 summarizes the demographics and clinical data of the patients.

### 3.2. Supracrestal Tissue Height (STH) Changes

The overall mean initial STH (STH1) was 2.15 mm (SD = 0.30 mm) and the mean final STH (STH2) was 3.06 mm (SD = 0.35 mm), resulting in an average STH gain of 0.91 mm (SD = 0.37 mm).

### 3.3. Gender Comparison

A comparison of STH gains between males and females showed no significant differences:

1. Males: mean STH gain = 0.88 mm (SD = 0.36 mm)
2. Females: mean STH gain = 0.93 mm (SD = 0.39 mm)
3. *t*-test: *t* = −0.305, *p*=0.763.

### 3.4. Influence of Additional Surgical Procedures

The study investigated the impact of different additional surgical procedures and biomaterials on STH gain. An ANOVA test revealed no significant differences between the groups:

1. ANOVA: *F* = 0.877, *p*=0.579.

### 3.5. Correlation Analysis

Correlation analyses were conducted to assess the relationship between STH gain and variables such as age, number of layers, and days to biopsy:

1. Age: *r* = −0.088, *p*=0.662 (no significant correlation)
2. Number of layers: *r* = 0.746, *p*=8.078*e* − 06 (significant positive correlation)
3. Days to biopsy: *r* = −0.165, *p*=0.410 (no significant correlation).

### 3.6. Summary of Key Findings

1. Gender: no significant differences in STH gain between males and females
2. Additional procedures: no significant impact on STH gain from different surgical procedures and biomaterials
3. Layers: a significant positive correlation between the number of layers used and STH gain
4. Age and days to biopsy: no significant correlations with STH gain.

### 3.7. Clinical Findings

Macroscopically clinically, the wound healing process was unremarkable in all patients. All implants could be restored in the previously planned manner. The follow-up period was up to 3 years from the first surgery and up to 2.5 years after prosthetic loading. The implant survival was 100% and all implants belong to implant quality scale group I after implant success criteria following the Pisa consensus conference [48]. There was no implant transparency in any of these cases.

### 3.8. Histological Analysis

#### 3.8.1. Healthy Mucosa (Control)

**Figure 13:**
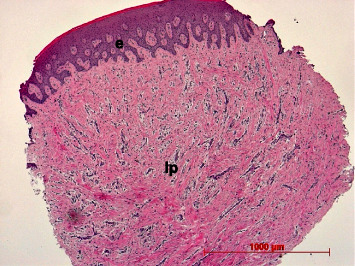
Overview of mucosal biopsy specimen showing gingival epithelium (e) and lamina propria (lp); case 3, H.E., original magnification ×5.

A regular oral mucosal structure was found showing an ortho- or parakeratinized stratified squamous epithelium with epithelial ridges and a subepithelial vascularized lamina propria. The lamina propria consisted of collagen fibers arranged in coarse irregular interwoven bundles, fibroblasts, vessels, and nerves. A superficial loosely arranged papillary layer could be differentiated from a deep reticular layer with thick, parallel bundles of collagen fibers (Figure 13).

#### 3.8.2. Augmented Mucosa

**Figure 14:**
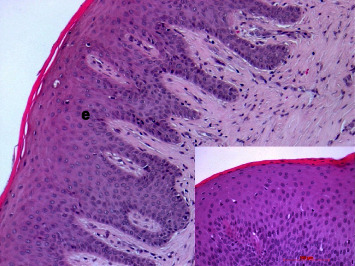
Keratinized gingival stratified squamous epithelium (e) with epithelial ridges; case 16, H.E., original magnification ×20; Inset: parakeratinized epithelium (above); case 9, H.E., original magnification ×40.

**Figure 15:**
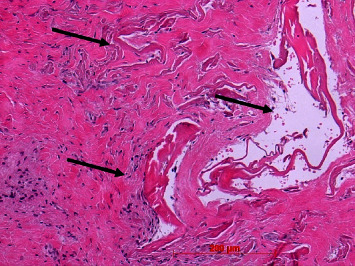
Larger Novomatrix® remnants (arrows) in deeper layer of lamina propria; case 18, H.E., original magnification ×20.

A regular oral mucosal stratified squamous epithelium could be found in all biopsies. In nearly all cases, the epithelium was parakeratinized. Epithelial ridges were of different diameters and depth but did not show apical proliferation in all cases (Figures 13 and 14). The lamina propria resembled the collagenous structure and composition of the healthy mucosa (Figure 14). Only in a few cases, remnants of the membrane could be identified as longish, amorphous eosinophilic strands intermingled among collagenous fibers in the reticular layer. In one case (no. 18), larger remnants could be found (Figure 15).

**Figure 16:**
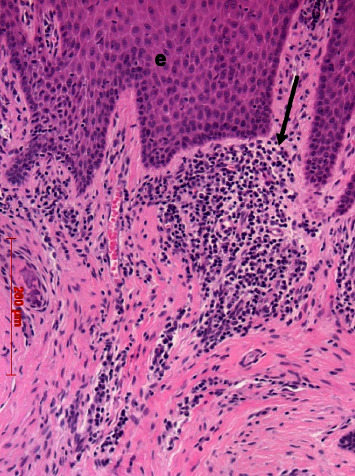
Subepithelial loosely packed round cell infiltrations (arrow): case 22, H.E., original magnification ×20.

**Figure 17:**
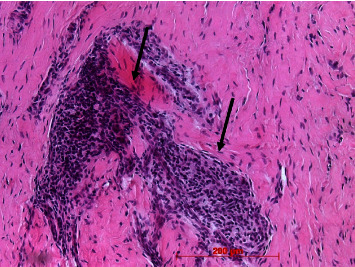
Larger dense infiltration (arrows) in the lamina propria; case 6, H.E., original magnification ×20.

**Figure 18:**
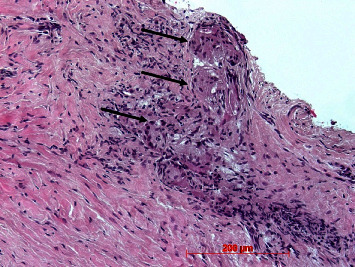
Infiltration in the apical region of the lamina propria with multinucleated foreign body giant cells (arrows); case 7, H.E., original magnification ×20.

Small hyaline bodies and granuloma formation were found in four cases. In all specimens, infiltrations could be found. In most cases, infiltrations were small or loosely arranged and consisted of round cells and macrophages. They were localized subepithelially in the papillary layer, pervascularly or deep in the reticular layer (Figures 16–18).

In five cases, the infiltrations were dense, of larger extension or formed the abovementioned granuloma. In two cases, small groups of multinucleated foreign body cells appeared (Figure 18). In cases with inflammation, no membrane residues could be detected. In half of the cases, remnants of bone substitutes, e.g., allogenic or autogenous dentin granules were observed in the deep layers (data not shown).

### 3.9. Immunohistochemistry

#### 3.9.1. Collagen Type I

**Figure 19:**
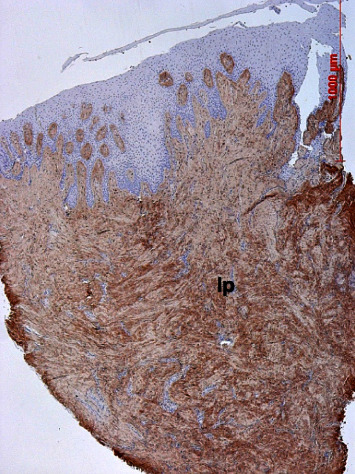
Immunostaining (brown color) for collagen type I in the lamina propria (lp), weaker staining in the papillary layer, and moderate staining in the apical reticular layer; case 4, DAB, original magnification ×5.

Immunostaining was similar between the control and test specimens and revealed weak to moderate staining of fiber bundles all over the lamina propria (Figure 19).

#### 3.9.2. Osteopontin

**Figure 20:**
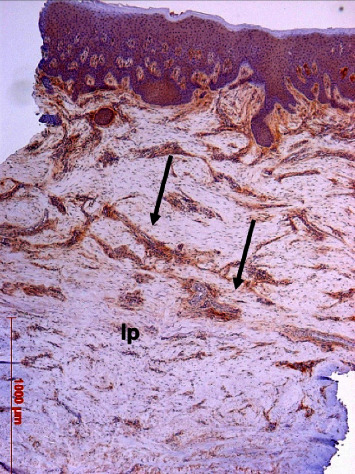
Immunostaining (brown color) for osteopontin in the lamina propria (lp), mainly in the subepithelial papillary layer and around vessels (arrows); case 21, DAB, original magnification ×5.

A weak to moderate immunoreactivity was found mainly in the perivascular fibrous tissue and in the papillary layer with no differences between control and test biopsies (Figure 20).

#### 3.9.3. vWF

**Figure 21:**
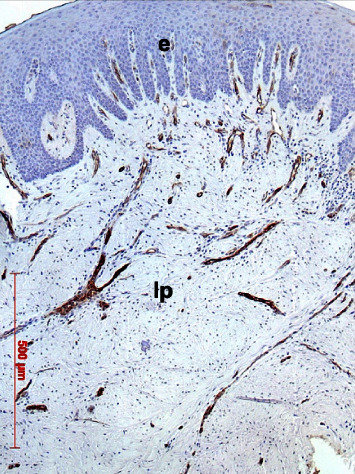
Immunostaining (brown color) for vWF to demonstrate vascularization, regular and moderate vascularization throughout the lamina propria (lp), *e* = epithelium; case 5, DAB, original magnification ×10.

Immunostained vessel walls belonged to arterioles and venoles located in the deep layer and a finer reticular vessel network located in the papillary layer in all specimens investigated (Figure 21). In the augmented mucosae, no avascular or hypervascularized areas could be observed.

## 4. Discussion

This case series aimed to evaluate the clinical efficacy of a novel prehydrated, porcine-derived acellular dermal matrix graft to thicken the peri-implant soft tissues at the time of implant placement in a private dental practice. An adequate soft tissue volume around dental implants appears to be one of the most important factors for peri-implant health and favorable esthetic outcomes [36, 49]. Implant sites lacking a suitable amount of soft tissue volume or KMW (keratinized mucosa width) can be improved by soft tissue augmentation procedures to prevent implant-related complications or esthetic drawbacks. Utilization of autologous tissues can still be considered as the gold standard for both procedures [49–51]; however, donor site morbidity, increased surgical time, discoloration, and a limited amount of donor site tissue can be seen as disadvantages [52, 53]. Interestingly, soft tissue thickening procedures do not seem to automatically lead to an increase in keratinized tissue, regardless of the biomaterial used [36].

Establishment of keratinized mucosa and soft tissue thickening should therefore be addressed as separate problems. In the patient cohort, the selection criteria ensured that there was an adequate zone of keratinized and attached mucosa surrounding the implants [53].

A reasonable threshold of STH for long-term implant success appears to be approximately 3 mm [10, 21, 24]. Patients enrolled in this study were characterized in the first place by a STH ≤2.5 mm and patient preference for a lower morbidity of the surgical intervention. Acellular dermal matrix grafts are a well-documented alternative to CTGs for plastic periodontal surgery in terms of recession reduction, soft tissue volume gain, and aesthetic appearance, but they seem to underperform in KMW gain around teeth [54].

ADMG in combination with implant surgery procedures indicates comparable results with CTGs but data are still very limited [51, 54]. This underscores the need for early and honest patient education to enable participatory decision-making. An increase in STH was achieved in all patients. Two layers of PPADMG resulted in more STH gain than one single layer. ADMGs are known for their technique sensitivity [55]; therefore, a tension-free surgical site coverage to avoid consecutive dehiscence at lower risk was preferred to maximum soft tissue augmentation, especially in combination with GBR procedures. One possible reason for the technique sensitivity could be due to the following: H&E-stained sections of PPADMG demonstrated densely packed collagen fibers in comparison with CTG. They show a significantly increased maximum load in tensile strength measurements compared to CTG [56]. The integration of the PPADMG might therefore be prolonged compared to CTG, since its degradation may take a longer period than that of a comparable CTG. At this time, no evidence-based statement can be made regarding how many layers of PPADMG on top of each other can integrate predictably.

The postoperative pain management was carried out according to the standard recommendations [57]. Nevertheless, there is currently no clear recommendation for the use of pre- and postoperative antibiotics in connection with augmentation procedures around dental implants [58]. Even for implantation without bone or soft tissue augmentation, the literature appears contradictory [59, 60].

All implants were restored as previously planned, and follow-ups were unremarkable, but the follow-up period is limited. Of the 27 specimens, only four were without additional surgical intervention except soft tissue augmentation with PPADMG. Thirteen specimens were combined with autologous bone or dentin augmentation, and ten specimens had additional allogeneic and xenogeneic materials used additionally to the PPADMG. The use of the absorbable suture material, which was intended to ensure positional stability of the PPADMG, may also have resulted in residuals [61]. This complicates the interpretation of the results, as well as different times to biopsy. The specimen with the shortest time to biopsy of 61 days showed only minimal membrane residues. It is therefore likely that the integration of the PPADMG will not take significantly longer than 2 months, even with two-layer application.

The histological investigations showed that in general the structure and composition of the augmented mucosa resembles those of the healthy oral mucosa in comparison to the control specimens. This includes the structure of an ortho- or parakeratinized gingival epithelium and a vascularized fibrous collagenous lamina propria containing collagen type I, which is the main collagen type of this connective tissue layer [62].

Immunohistochemically, also osteopontin could be detected, which is an abundant noncollagenous, nonspecific protein appearing in connective tissues and involved in wound healing and angiogenesis [63]. It can be speculated that the remodeling of the subepithelial matrix could be triggered by the membrane applied. It is known that this acellular dermal matrix can repopulate fibroblasts responsible for matrix turnover and adsorb and release growth factors involved in regeneration processes [64–66]. The biopsies of the test group showed a good subepithelial vascularization as visualized immunohistochemically by using the von Willebrand factor as a vessel marker. This could be the result of vasculogenesis triggered by the membrane and enabled by its porosity. As formerly investigated in animal models, porcine dermal-derived collagenous membranes are characterized by the ability to allow and stimulate angiogenesis [67, 68]. In our study, remnants of the membrane appear as eosinophilic or hyalinized structures resembling the typical residues as described for resorbable collagenous membranes [69]. Obviously, inflammation was related to complete degradation of the membranes. However, inflammation was mild and is a typical phenomenon during membrane degradation [70]. In two cases, multinucleated foreign body giant cells could be detected. It is discussed that these cells, when appearing in a moderate number, also may participate in the degradation of membranes and may have no negative impact on regeneration or healing. It also cannot be excluded that the multinucleated foreign body giant cells found could be caused by degradation of the absorbable sutures [70]. A larger number of specimens without further bone augmentative procedures would have been very helpful and would have facilitated the interpretation of the results. Currently available porcine acellular dermal matrix grafts differ in their processing procedures. The PPADMG investigated here does not use dehydration in the manufacturing process. This could explain the increased mechanical properties [56] and the superiority in an animal histology study regarding root coverage and increase in tissue thickness compared to another available porcine dermal matrix graft [71].

In particular, because of the small sample size, the heterogeneity of the augmentation procedures, the lack of a control group, and the potential selection bias, further randomized controlled trials and clinically controlled trials are needed to investigate the long-term outcomes of implant sites augmented with PPADMG in terms of peri-implant tissue health and volume stability. Nevertheless, the histological results show that it can be expected that after about 8 weeks the integration of the matrices will be completed without any particular tissue reactions. All implant sites showed an increase in STH, which was higher when two layers were used than when one layer was used. The application of PPADMG can present a challenge for inexperienced users, which can be overcome through adequate preparation such as training under the supervision of an experienced practitioner using appropriate models. The practically unlimited availability of PPADMG, compared to autologous soft tissues, also allows for the treatment of larger surgical sites or multiple quadrants in a single session. In terms of cost-effectiveness, it is difficult to make a definitive statement. Compared to the use of autologous tissues, additional material costs are incurred. However, these costs may be partially offset by shorter operation times and the lack of necessity for creating wound dressing plates. Generalization is challenging and depends on the specific clinical setting.

In comparison with autologous grafts, the following statements seem highly likely:

1. CTGs can still be considered the gold standard for tissue thickening around implants and teeth.
2. ADMGs have an advantage over CTGs in terms of comorbidity and the unlimited availability of the material in everyday clinical practice.
3. FSTs can still be considered the gold standard for the creation of keratinized tissues around implants.
4. Based on the histological findings of this study, ADMGs seem to require a longer healing period than some less dense CMs, especially when it comes to open healing. Therefore, the open healing of PPADMG does not appear to be the ideal indication for creating a zone of keratinized gingiva around implants [72].

There is little evidence comparing PPADMG and nonprehydrated porcine ADMGs. However, in preclinical cell experiments on wound healing and proliferation of oral fibroblasts and periodontal ligament cells, as well as in mechanical properties, the prehydrated form of porcine ADMG seems to have an advantage over the lyophilized form [9, 14, 17]. Whether this makes a clinical difference cannot yet be verified.

In addition to the discussed indications for PPADMG soft tissue thickening around implants and recession coverage of implants, the following applications seem promising:

1. Soft tissue thickening in the area of pontics for aesthetic improvement
2. Soft tissue modification in the context of orthodontic treatments
3. Creation of autologous connective tissue volume in easily operable areas after augmentation with PPADMG, which can then be autotransplanted at a later time and should be fully transformed after about eight weeks.
4. Use of PPADMG as a barrier membrane in conjunction with GBR procedures, where a shorter healing period is expected, such as horizontal GBRs with an augmentation width of up to 4 mm.

However, further studies are needed for these applications before routine clinical use can be considered safe and sensible.

## 5. Conclusions

Within the limitations of this case series with histological and clinical evaluation, it may be concluded that the use of this novel PPADMG at the time of implant placement and in combination with primary wound closure is a reliable and safe alternative to autologous connective tissue grafts to increase peri-implant STH.

## Figures and Tables

**Table 1:** Antibody details and incubation protocols for immunohistochemistry. A synoptic summary of the cases is given in Table 2.

Antibody	Isotype	Manufacturer	Incubation protocol
Collagen type I	Rabbit monoclonal	Abcam (Cambridge, UK)	1 : 400, 1 h, RT
Osteopontin	Rabbit polyclonal	Abcam (Cambridge, UK)	1 : 200, 1 h, RT
von Willebrand factor	Rabbit polyclonal	Linaris (Wertheim, Germany)	1 : 200, 1 h, RT

**Table 2:** Patient-related clinical data and histological analysis.

Patient	Gender	Age (years)	Site (ADA)	Layers	STH1 (mm)	Days to biopsy	STH2 (mm)	STH gain (mm)	Additional surgery or biomaterials	Histological results
1	m	58	#7	1	2	168	3	1	ARP dentin + Memlok RCM (107 d before STA	No residues and mild inflammation
1	m	58	#10	1	2	168	3	1	ARP dentin + Memlok RCM (107 d before STA)	No residues and mild inflammation
2	m	61	#22	1	2	96	2.5	0.5	None	No residues and mild inflammation
2	m	61	#27	1	1.5	96	2.5	1	GBR AB	Residues and mild inflammation
3	f	65	#19	1	2.5	180	3	0.5	None	No residues and inflammation-free
3	f	65	#20	1	2.5	268	3	0.5	GBR AB	No residues and inflammation-free
4	f	33	#29	2	2	61	3	1	None	Possible residues and inflammation-free
4	f	33	#30	2	2	93	3	1	GBR AB + MinerOss X	No residues and mild inflammation
5	f	77	#22	2	2	182	3.5	1.5	GBR dentin	No residues and mild inflammation
5	f	77	#27	2	2	182	3.5	1.5	GBR dentin	No residues and mild inflammation
6	f	51	#29	1	2.5	110	3	0.5	GBR AB	Residues, foreign body reaction, and moderate inflammation
7	f	66	#19	1	2	126	3	1	GBR AB	Residues, foreign body reaction, and moderate inflammation
8	m	75	#8	1	2.5	100	3	0.5	GBR MinerOss X + Memlok RCM	Residues and mild inflammation
9	f	62	#28	1	2.5	151	3	0.5	GBR AB + Maxgraft CCG + Memlok RCM	Residues and mild inflammation
10	m	64	#30	1	2.5	142	3	0.5	GBR dentin	No residues and minimal inflammation
11	m	76	#21	2	2	66	3	1	GBR AB	No residues and mild inflammation
12	f	54	#19	1	2	240	3	1	GBR AB	No residues and minimal inflammation
13	f	62	#30	1	2	108	2.5	0.5	ARP MinerOss X + Memlok RCM (190 d before STA), GBR with bone from ARP-site	No residues and minimal inflammation
14	m	61	#29	2	2,5	164	3.5	1	GBR MinerOss X + Ossix plus	Residues and mild inflammation
15	m	55	#18	2	2	133	3.5	1.5	GBR AB	No residues and mild inflammation
16	f	65	#29	2	2	121	3	1	GBR AB	No residues and moderate inflammation
17	f	31	#4	2	2.5	162	4	1.5	GBR AB	No residues and inflammation-free
18	m	56	#18	2	2	143	3.5	1.5	ARP MinerOss X + Jason fleece (292 d before STA), GBR with bone from ARP-site + Memlok RCM	Residues and minimal inflammation
19	f	52	#30	2	2	176	3	1	GBR AB + Volumax (138d before STA), GBR with bone from ARP-site	Residues and minimal inflammation
20	m	57	#18	1	2.5	227	3	0.5	GBR AB	No residues and minimal inflammation
21	m	52	#19	2	1.5	127	2.5	1	None	No residues and minimal inflammation
22	m	46	#8	1	2.5	445	3	0.5	GBR MinerOss X + Memlok RCM	No residues and minimal inflammation

## Data Availability

The data that support the findings of this study are available from the corresponding author upon reasonable request.

## Abbreviations

ADMG: Acellular dermal matrix grafts
ARP: Alveolar ridge preservation
APF: Apically positioned flap
AB: Autologous bone
BSM: Bone substitute materials
BSA: Bovine serum albumin
CM: Collagen membranes
COL1: Collagen type I
CTG: Connective tissue grafts
DAB: Diaminobenzidine
EDTA: Ethylenediaminetetraacetic acid
FGG: Free gingival graft
GBR: Guided bone regeneration
GBR: Guided bone regeneration
HE: Hematoxylin-eosin
KMW: Keratinized mucosa width
KPIM: Keratinized peri-implant mucosa
MBL: Marginal bone loss
OP: Osteopontin
PAS: Periodic acid Schiff
PES: Pink esthetic scores
PPADMG: Prehydrated porcine acellular dermal matrix graft
RT: Room temperature
STA: Soft tissue augmentation
STH: Supracrestal soft tissue height
TBS: Tris-buffered saline
vWF: von Willebrand factor.
